# Obesity and outcomes in patients undergoing upper airway surgery for obstructive sleep apnea

**DOI:** 10.1371/journal.pone.0272331

**Published:** 2022-08-11

**Authors:** Austin L. Du, Jeffrey L. Tully, Brian P. Curran, Rodney A. Gabriel

**Affiliations:** 1 School of Medicine, University of California, San Diego, La Jolla, California, United States of America; 2 Department of Anesthesiology, Division of Perioperative Informatics, University of California, San Diego, La Jolla, California, United States of America; 3 Department of Medicine, Division of Biomedical Informatics, University of California, San Diego, La Jolla, California, United States of America; Sapienza University of Rome, ITALY

## Abstract

**Objective:**

Obesity is frequently debated as a factor associated with increased postoperative complications. Specifically, upper airway surgeries for obstructive sleep apnea (OSA), a common comorbidity among obese patients, may be complicated by obesity’s impact on intraoperative ventilation. The aim of this retrospective study was to analyze the association of various degrees of obesity with postoperative outcomes in patients undergoing surgery for OSA.

**Methods:**

The American College of Surgeons National Surgical Quality Improvement database between 2015 and 2019 was used to create a sample of patients diagnosed with OSA who underwent uvulopalatopharyngoplasty, tracheotomy, and surgeries at the base of tongue, maxilla, palate, or nose/turbinate. Inverse probability-weighted logistic regression and unadjusted multivariable logistic regression were used to compare outcomes of non-obese and obesity class 1, class 2, and class 3 groups (World Health Organization classification). Primary outcome was a composite of 30-day readmissions, reoperations, and/or postoperative complications, and a secondary outcome was all-cause same-day hospital admission.

**Results:**

There were 1929 airway surgeries identified. The inverse probability-weighted regression comparing class 1, class 2, and class 3 obesity groups to non-obese patients showed no association between obesity and composite outcome and no association between obesity and hospital admission (all p-values > 0.05).

**Conclusion:**

These results do not provide evidence that obesity is associated with poorer outcomes or hospital admission surrounding upper airway surgery for OSA. While these data points towards the safety of upper airway surgery in obese patients with OSA, larger prospective studies will aid in elucidating the impact of obesity.

## Introduction

The World Health Organization (WHO) defines overweight and obese as abnormal or excessive fat accumulation that presents a risk to health [1]. In the United States, from 1999 to 2018, obesity prevalence increased from 30.5% to 42.4%, with class 3 obesity (body mass index [BMI] ≥40 kg/m^2^) increasing from 4.7% to 9.2% [2]. Recent data have identified obesity as an important risk factor and disease modifier of obstructive sleep apnea (OSA) due to its direct effect on nocturnal respiratory effort [3]. OSA and obesity may also exhibit two-way causality due to their reciprocal effects on oxidative stress, systemic inflammation, and the intestinal microbiota. Independently, OSA is recognized as a risk factor for conditions such as hypertension, stroke, depression, and diabetes [4]. When considering concurrent obesity and OSA, neurocognitive impairment represents a trait d’union, manifesting as depression, sleepiness and mood changes [5].

Upper airway surgery has emerged as an alternative to positive airway pressure for patients with severe OSA and may become more prevalent as more patients are diagnosed [6]. However, large observational trials have reported major cardiovascular and pulmonary complications in OSA patients requiring general anesthesia [7, 8], while OSA and obesity have been shown to have supraadditive effects on adverse outcomes following abdominal procedures [9]. Underreporting for both conditions may also underestimate the impact of these conditions on perioperative outcomes. Limited data on obesity and upper airway surgeries have associated elevated BMI with lower success rates [10] and increased likelihood of hospital admission [11] in patients undergoing tonsillectomies for obstructive sleep apnea.

Similar discussions surrounding the perioperative impact of OSA and obesity also ask whether outpatient surgery is appropriate for these patients. The Society for Ambulatory Anesthesia (SAMBA) consensus statement on preoperative selection of adult patients with OSA scheduled for ambulatory surgery did not provide any guidance on the safety of ambulatory upper airway surgery due to limited evidence [12], and the American Society of Anesthesiologists (ASA) does not recommend performing airway surgery on an outpatient basis [13]. Indeed, evidence has pointed towards the benefits of inpatient admission for some oral and maxillofacial procedures, especially given difficult airway management in some obese patients [14, 15]. The usage of BMI “cutoffs” for outpatient upper airway surgery is controversial [16].

Consensus statements recognize that data on upper airway surgery outcomes in OSA patients are sparse [17], and the role of obesity in postoperative outcomes of these patients is poorly understood. The aim of this study was to evaluate the association of obesity and postoperative outcomes following upper airway surgery for OSA. Our hypothesis was that obesity is associated with worse postoperative outcomes and increased incidence of same-day hospital admission.

## Materials and methods

### Data registry

This study was not considered human subjects research as we utilized a public de-identified database, and thus the study was exempt from consent requirements by our Institutional Review Board (Human Research Protection Program). Data from the American College of Surgeons National Surgical Quality Improvement (ACS-NSQIP) databases between January 1, 2015 and December 31, 2019 were used. In generating the NSQIP database, Surgical Clinical Reviewers extract the data from patients’ chart, and interrater reliability audits are conducted regularly to assess the quality of the data collected [18]. The most recent iteration of ACS-NSQIP includes over 1 million cases and 700 participating United States hospitals. This manuscript adheres to the Strengthening the Reporting of Observational Studies in Epidemiology (STROBE) and Enhancing the Quality and Transparency of Health Research (EQUATOR) guidelines.

### Study population and outcome measures

Adult patients with obstructive sleep disorders were identified using International Classification of Diseases, Tenth Revision, Clinical Modification (ICD-10-CM) diagnosis codes G47.30 (sleep apnea, unspecified), G47.33 (obstructive sleep apnea, adult), and G47.39 (other sleep apnea). Airway surgeries for treatment of OSA were also identified using current procedural terminology (CPT) codes (S1 Appendix) and categorized by anatomic location (nasal or sinus surgery; uvulopalatopharyngoplasty [UPPP], tonsillectomy, or other palate surgery; base of tongue procedures; maxillomandibular advancement; and tracheostomy). To create a homogenous sample of elective cases, exclusion criteria included inpatients at acute care hospitals; patients at nursing homes, chronic care, or intermediate care facilities; emergency cases and nonelective cases.

Patient demographics (age, sex, race), comorbidities (diabetes mellitus, chronic obstructive pulmonary disorder [COPD], hypertension, bleeding disorders), other clinical characteristics (chronic steroid use, smoking history, dyspnea, dialysis, ASA physical status, and functional status), and surgical complexity were collected among all patients included. Surgical complexity was evaluated by measuring the sum of relative value units (RVUs) for the primary and secondary procedures [19], the total number of concurrent procedures, total operation time, and whether the procedure was performed on multiple levels. For example, single-level procedures included UPPP alone while multilevel surgery included combinations of UPPP and surgery in another anatomic location.

The primary outcome of interest for the study was a composite measure of all-cause readmission to the same or different hospital, reoperation, or postoperative complication (surgical site infection, dehiscence, pneumonia, reintubation, pulmonary embolism, acute renal failure, stroke, cardiac arrest, myocardial infarction, bleeding requiring transfusion, deep vein thrombosis, sepsis, or death) within 30 days of the primary surgical procedure. Secondary outcome of interest was same-day hospital admission. Of note, hospitalizations in the NSQIP database are not specific to “planned” or “unplanned” admissions, but instead represent any cases performed in an inpatient setting [20]. A surgery was classified as an admission if the length of stay was 1 day or longer. BMI was our primary exposure variable, with cohorts split into four BMI ranges according to WHO classifications [1]. BMI defined as non-obese was used as our reference cohort:

1. Non-obese: <30 kg/m^2^ (reference cohort)
2. Class 1 Obesity: ≥30 and <35 kg/m^2^
3. Class 2 Obesity: ≥35 and <40 kg/m^2^
4. Class 3 Obesity: ≥40 kg/m^2^

### Statistical analysis

R (version 4.1.2) was the statistical computing software used to perform all statistical analyses. Patients were split into four BMI cohorts as outlined previously. Baseline recipient characteristics were summarized according to data at the time of surgery and presented as percentages for categorical variables and mean ± standard deviation (SD) for continuous variables. Analysis of variance (ANOVA) and chi-square tests were used to compare continuous and categorical characteristics, respectively, among all four BMI cohorts.

To address systematic differences between obese and non-obese patients, primary and secondary outcomes of interest were each measured using inverse probability-weighted (IPW) logistic regressions. We used generalized boosted models to generate a model that estimated propensity scores across each BMI cohort. This model predicted the probability of each patient being in each of the four BMI cohorts [21, 22]. The balance in covariates across all four BMI cohorts was evaluated using maximum standardized mean differences (SMDs), with any SMDs greater than 0.1 indicating covariate imbalance [23]. These probabilities were then used to apply weights to each patient during regression analyses, which allowed us to include all patients and outcome events in our analysis. The final univariate IPW regressions measured the relative odds of each outcome among obese (Class 1, Class 2, or Class 3) compared to non-obese patients. Covariates fed into the propensity score model included all variables mentioned in the previous section, namely patient demographics, comorbidities, measures of surgical complexity, and other clinical characteristics.

In addition, we performed a multivariable logistic regression measuring the association of obesity class 1, 2, or 3 on odds of the composite outcome and hospital admission compared to non-obese patients. In this analysis, all confounding variables which showed significant differences (p < 0.05) in either ANOVA or chi-square analyses were included as covariates. The odds ratio (OR) and 95% confidence interval (CI) were presented and p-values less than 0.05 were considered statistically significant for all outcomes.

## Results

A total of 1929 airway surgeries were identified (Table 1). Patients with class 3 obesity demonstrated the highest prevalence of female sex, diabetes, hypertension, tobacco use, dyspnea, and poor ASA physical status. Total RVUs, number of procedures, and total procedure time did not differ significantly between obesity groups. UPPP was performed in 97% of patients having surgery, while nasal surgical procedures were the most common concomitant procedure, occurring concurrently in 26.9% of UPPP procedures.

**Table 1:** Baseline characteristics of the study population.

	Non-obese	Class 1 Obese	Class 2 Obese	Class 3 Obese	P-value
n	708	608	322	291	
Age (mean (SD))	39.75 (13.66)	41.72 (12.98)	41.21 (12.02)	39.03 (11.49)	0.006
Male (%)	536 (75.7)	471 (77.5)	226 (70.2)	159 (54.6)	<0.001
Race (%)					<0.001
White	403 (56.9)	365 (60.0)	197 (61.2)	174 (59.8)	
American Indian or Alaska Native	1 (0.1)	1 (0.2)	3 (0.9)	2 (0.7)	
Asian	67 (9.5)	35 (5.8)	13 (4.0)	7 (2.4)	
Black or African American	63 (8.9)	87 (14.3)	41 (12.7)	66 (22.7)	
Native Hawaiian or Pacific Islander	8 (1.1)	7 (1.2)	6 (1.9)	6 (2.1)	
Unknown/Not Reported	166 (23.4)	113 (18.6)	62 (19.3)	36 (12.4)	
ASA Physical Status (%)					<0.001
1	61 (8.6)	19 (3.1)	6 (1.9)	2 (0.7)	
2	493 (69.6)	386 (63.5)	153 (47.5)	66 (22.7)	
3	153 (21.6)	198 (32.6)	163 (50.6)	209 (71.8)	
4	1 (0.1)	5 (0.8)	0 (0.0)	14 (4.8)	
Diabetes (%)	31 (4.4)	52 (8.6)	51 (15.8)	63 (21.6)	<0.001
COPD (%)	3 (0.4)	11 (1.8)	5 (1.6)	3 (1.0)	0.104
Hypertension (%)	136 (19.2)	171 (28.1)	124 (38.5)	124 (42.6)	<0.001
Chronic Steroids (%)	11 (1.6)	7 (1.2)	6 (1.9)	9 (3.1)	0.205
Bleeding Disorder (%)	4 (0.6)	2 (0.3)	1 (0.3)	2 (0.7)	0.833
Smoker (%)	80 (11.3)	74 (12.2)	47 (14.6)	58 (19.9)	0.002
Dyspnea (%)	13 (1.8)	23 (3.8)	11 (3.4)	16 (5.5)	0.021
Partially or Totally Dependent Functional Status (%)	2 (0.3)	0 (0.0)	2 (0.6)	3 (1.0)	0.089
On Dialysis (%)	2 (0.3)	1 (0.2)	0 (0.0)	0 (0.0)	0.638
UPPP (%)	686 (96.9)	589 (96.9)	314 (97.5)	283 (97.3)	0.938
Nasal or Sinus (%)	183 (25.8)	163 (26.8)	86 (26.7)	83 (28.5)	0.859
Other Palate (%)	15 (2.1)	17 (2.8)	8 (2.5)	3 (1.0)	0.399
Base of Tongue (%)	75 (10.6)	52 (8.6)	39 (12.1)	34 (11.7)	0.285
Maxillomandibular Advancement (%)	10 (1.4)	6 (1.0)	4 (1.2)	4 (1.4)	0.912
Tracheostomy (%)	0 (0.0)	0 (0.0)	0 (0.0)	1 (0.3)	0.131
Multi-level Surgery (%)	237 (33.5)	199 (32.7)	114 (35.4)	105 (36.1)	0.713
Total RVUs (mean (SD))	13.11 (8.16)	13.28 (8.28)	13.36 (7.96)	12.81 (8.07)	0.83
Total Procedures (mean (SD))	1.99 (1.14)	2.02 (1.16)	2.01 (1.11)	1.98 (1.05)	0.936
Operating Time (mean (SD))	62.23 (45.39)	64.83 (54.33)	62.90 (43.26)	66.89 (56.06)	0.533

Percentages for categorical variables and mean ± standard deviation (SD) for continuous variables are presented. Analysis of variance (ANOVA) and chi-square tests were used to compare continuous and categorical characteristics, respectively. Abbreviations: SD, standard deviation; UPPP, uvulopalatopharyngoplasty; ASA, American Society of Anesthesiologists; COPD, chronic obstructive pulmonary disorder; ANOVA, Analysis of variance; RVU, relative value unit

**Fig 1:**
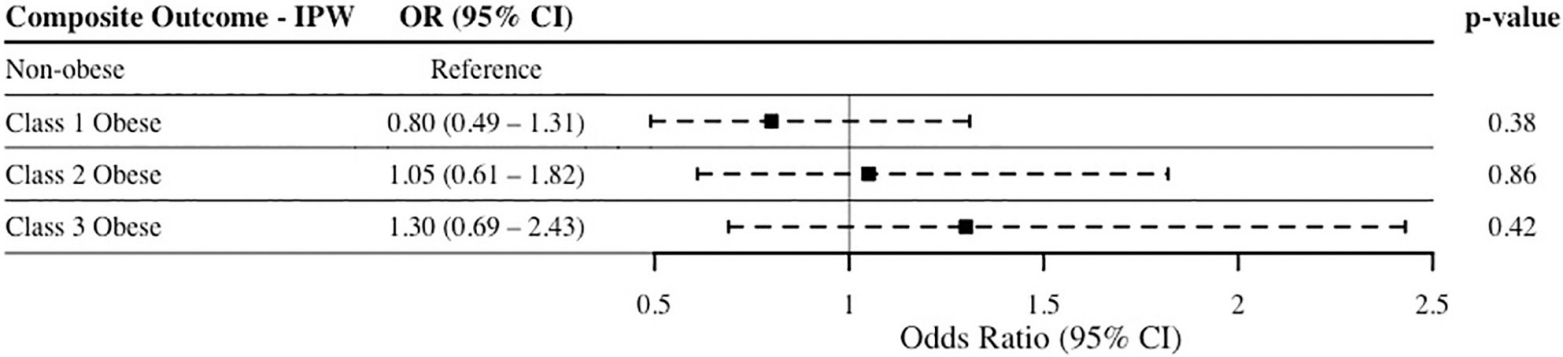
IPW-adjusted logistic regression of composite outcome. Composite outcome represents readmission, reoperation, or complications. Outcome regressed on obesity categories, with non-obese (BMI <30 kg/m^2^) patients used as the reference group. Abbreviations: IPW, inverse probability-weighing; OR, odds ratio; CI, confidence interval.

**Fig 2:**
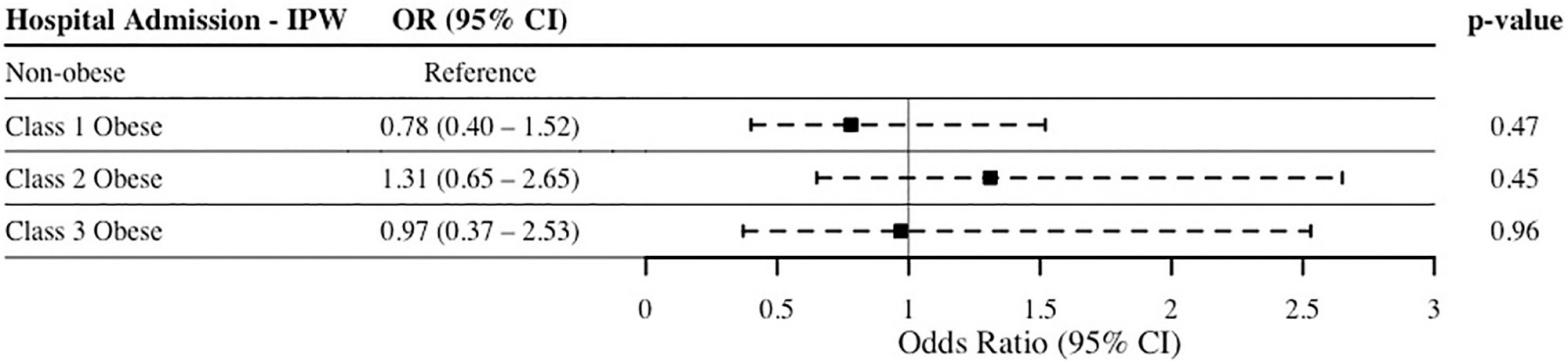
IPW-adjusted logistic regression of all-cause hospital admission. Outcome regressed on obesity categories, with non-obese (BMI <30 kg/m^2^) patients used as the reference group. Abbreviations: Abbreviations: IPW, inverse probability-weighing; OR, odds ratio; CI, confidence interval.

The overall rate of hospital admission across all groups was 61.9%, and the composite rate of readmissions, reoperations, and/or complications in the whole unmatched sample was 6.5%. An IPW-adjusted regression of both hospital admission and composite outcome of readmission, reoperation, or complications was conducted. SMDs for each covariate were well-balanced in the final matched sample, i.e. the absolute standardized difference in covariate value between all groups was < 0.1 across all covariates. Fig 1 represents the IPW-adjusted regression of composite outcome comparing non-obesity and class 1, 2, and 3 obesity, while Fig 2 represents the IPW-adjusted regression of hospital admissions of non-obesity and class 1, 2, and 3 obesity. Compared to non-obese patients, class 1 (p = 0.20), class 2 (p = 0.87), and class 3 (p = 0.90) obesity were not associated with significantly different odds of the composite outcome of readmission, reoperation, or complications. Similarly, class 1 (p = 0.47), class 2 (p = 0.45), and class 3 (p = 0.96) obesity were not associated with significantly different odds of 30-day hospital admission compared to non-obese patients.

**Fig 3:**
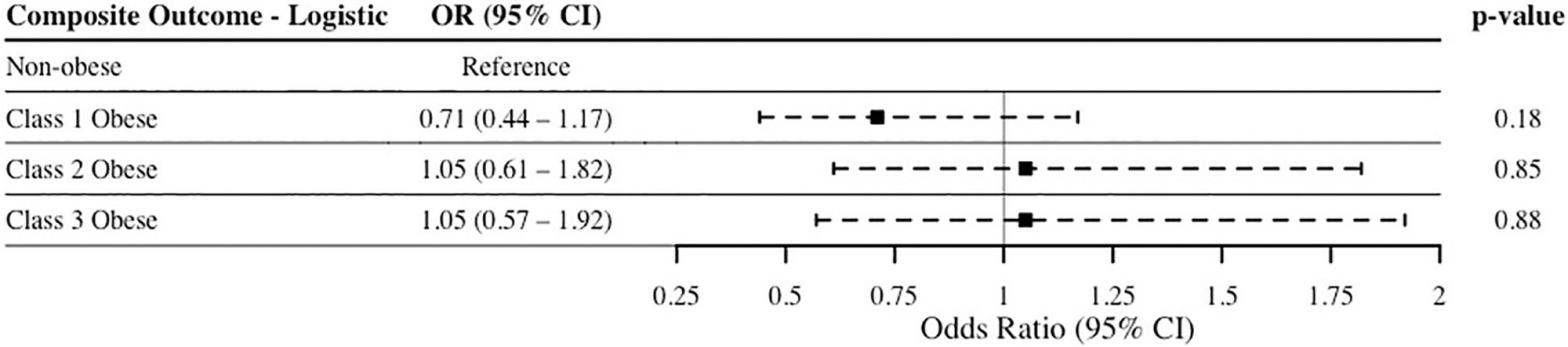
Unadjusted multivariable logistic regression of composite outcome. Composite outcome represents readmission, reoperation, or complications. Outcome regressed on obesity categories, with non-obese (BMI <30 kg/m^2^) patients used as the reference group. Abbreviations: OR, odds ratio; CI, confidence interval.

**Fig 4:**
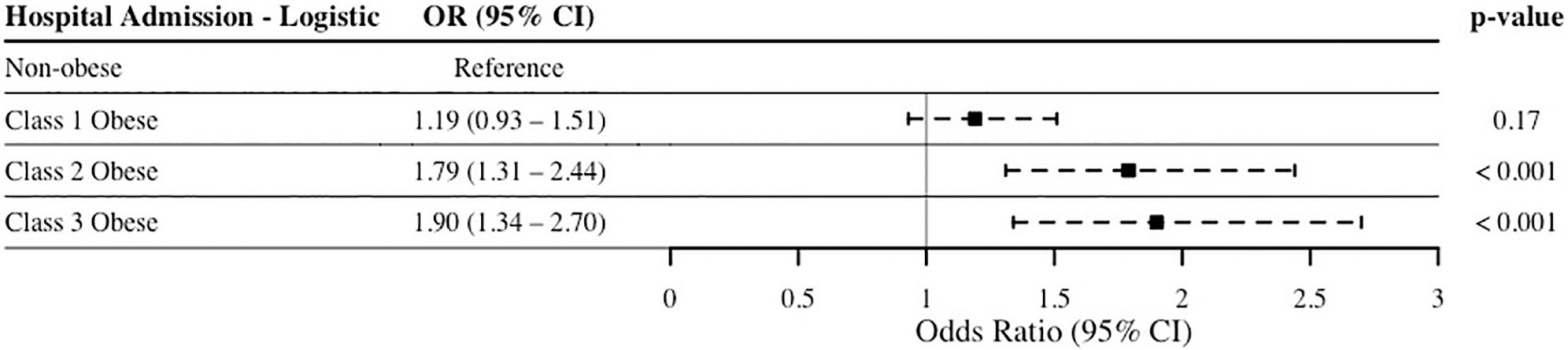
Unadjusted multivariable logistic regression of all-cause hospital admission. Outcome regressed on obesity categories, with non-obese (BMI <30 kg/m^2^) used as the reference group. Abbreviations: OR, odds ratio; CI, confidence interval.

Unadjusted multivariable logistic regression models included age, sex, race, surgery, surgical complexity (i.e. RVUs, procedures, operating time), surgery type, and comorbidities (diabetes, hypertension, smoking status, and dyspnea) as covariates. Results of the regression of composite outcome (Fig 3) did not reveal a significant difference between non-obese patients and class 1 (p = 0.18), class 2 (p = 0.85), and class 3 (p = 0.88) obese patients. In contrast, both class 2 (OR 1.79, 95% CI 1.31–2.44, p<0.001) and class 3 obesity groups (OR 1.90, 95% CI 1.34–2.70, p<0.001) were associated with increased odds of hospital admission (Fig 4).

## Discussion

Obesity continues to be a growing area of concern for patients undergoing upper airway surgery for OSA. In IPW-adjusted and IPW and multivariable unadjusted logistic regressions, we did not find associations between obesity and postoperative outcomes, measured as a composite of readmission, reoperation, or complications. In parallel, we did not identify significant associations between obesity and all-cause hospital admission.

Obesity continues to increase in prevalence and is associated with comorbidities including hypertension, type II diabetes mellitus, dyslipidemia, sleep breathing disorders, some cancers and major cardiovascular disease [24]. Cardiovascular sequelae such as heart failure, coronary artery disease, sudden cardiac death and atrial fibrillation contributes to reduced overall survival among obese patients. Mechanisms contributing to this cardiac dysfunction include the direct production of pro-inflammatory cytokines by adipose tissue, promoting the formation of atherosclerotic plaques [25]. However, numerous studies, including the results presented here, have documented an “obesity paradox” in which obese patients demonstrate improved outcomes in response to stresses such as peripheral artery disease, stroke, and cardiac surgery, among others [26]. Although new studies continue to explore this topic, dominant hypotheses assert that BMI measurements are inherently confounded by calculations of lean body mass rather than adiposity [27]. Given that obese individuals typically present with an increased amount of lean mass in addition to fat mass, this may partially explain the paradox as lean mass is associated with improved cardiorespiratory fitness [25]. Cardiorespiratory fitness is a major determinant of clinical outcomes, particularly in those with cardiovascular diseases common in our study population. Alternative theories include comparatively large benefits of adjunct respiratory instrumentation [28] and anesthetics that preserve respiratory function [29]. Of course, excessive adipose deposits for many patients would counteract the benefits of elevated BMI. For instance, the obesity-associated “metabolic syndrome” consisting of hypertension, dyslipidemia, and type II diabetes mellitus is a risk factor for perioperative morbidity and mortality, warranting advanced care for obese patients undergoing general anesthesia [30]. Additional data reveal associations between obesity and surgical site infections [31], respiratory complications [32], and post-tonsillectomy hemorrhage [33]. The balance between increased muscle mass and excessive adipose tissue thus are two counteracting forces on the impact of BMI on the upper airway surgeries analyzed here. This creates a heterogenous population of high-BMI patients and may have contributed to our non-significant outcome.

Similar observations can explain the lack of association between obesity and hospital admissions in patients undergoing OSA surgery. Given the absence of an association with postoperative complications, our IPW-adjusted regression analysis is consistent with no difference in inpatient admission. However, this conclusion is complicated by our retrospective study design, as planned inpatient admissions for medically complicated cases are indistinguishable from admissions due to postoperative complications [20]. Nevertheless, these results contribute to a limited set of data exploring obesity as a consideration in deciding whether a procedure should be performed in an inpatient versus outpatient setting for OSA-related surgery. Elevated BMI is often evaluated as a contraindication for outpatient surgeries, although this is controversial [16]. As the prevalence of upper airway surgeries has increased in outpatient settings, patient selection criteria for these procedures has become increasingly debated [11]. Although inpatient admission is currently recommended for tonsillectomies in obese children [34], further studies in adults will elucidate the proper selection of adult patients for inpatient upper airway surgery. Among patients with contraindications, alternative procedures may provide similar benefits with lower risk of complications. For instance, barbed reposition pharyngoplasty offers greater stabilization of the pharyngeal lateral wall and while avoiding invasive resection of the soft tissues [35], while expansion sphincter pharyngoplasty has demonstrated promising long-term effectiveness with low morbidity and complication rates [36]. Encouraging data also exist regarding transoral robotic surgery as a means to reduce lingual tonsil volume and retrolingual collapse [37].

Our study is accompanied by limitations, primarily due to our retrospective study design. First, conclusions drawn here represent associations rather than causative effects of obesity. Specifically, some patients may have incurred some of the chronic conditions in Table 1 as a result, rather than a direct cause, of obesity. In these cases, a variable could be a potential mediator rather than a confounder [38]. The NSQIP registry also does not collect information regarding each patient’s severity of OSA. For instance, apnea-hypopnea index, oxygen nadirs, medications administered, and degree of oxygen desaturation are all metrics helpful in properly controlling for confounders in our analysis. Provider-driven errors in coding appropriate procedures and complications are also a common caveat in large database studies. Furthermore, a generally low incidence of life-threatening complications, paired with a relatively small representation in the NSQIP database contribute to decreased sample sizes. As mentioned previously, the database also does not include details regarding the exact circumstances of inpatient admissions, which would be beneficial in determining the exact cause of each admission.

## Conclusions

In summary, our results presented here do not support the conclusion that obesity is associated with poorer outcomes or hospital admission following upper airway surgery for OSA. While this points towards the safety of surgical treatment of OSA in obese patients, future studies will further confirm or refute this conclusion.

## Supporting information

S1 Appendix (DOCX)
