# Supplementary material for: Obesity and outcomes in patients undergoing upper airway surgery for obstructive sleep apnea
Source: PLoS One. 2022 Aug 11;17(8):e0272331. doi: 10.1371/journal.pone.0272331 (PMC9371252; doi:10.1371/journal.pone.0272331)
Supplement: S1 Appendix — (DOCX) [file pone.0272331.s001.docx]

| Surgery Category | Current Procedural Terminology codes |
| --- | --- |
| Uvulopalatopharyngoplasty, tonsillectomy, or adenoidectomy | 42145, 42950, 42140, 42821, 42826, 42831 |
| Nasal and sinus surgery | 30801, 30802, 30930, 30130, 30140, 30520, 30540, 30400, 30410, 30420, 30450, 30465, 31020, 31030, 31040, 31255, 31288, 31276, 31200, 31201 |
| Base of tongue surgery | 21199, 21120, 21121, 21122, 21123, 41120, 41130, 42870, 21685, 41512, 41530, 31420 |
| Maxillomandibular advancement | 21141, 21143, 21193, 21194, 21195, 21196, 21198, 21199, 21206 |
| Tracheostomy | 31600, 31610 |
| Other palate surgery | 42299, 42892, 42890, 42104, 42106, 42107, 42120, 42235 |
